# Prescriptions and Dispensing

**Published:** 1908-01-25

**Authors:** 


### THERAPEUTICS.

### PRESCRIPTIONS AND DISPENSING.

THE following may be regarded as a typical prescription for a simple emulsion:—

| | | |
|---|---|---|
| ℞ Olei Amygdalæ | ... | ʒiij. |
| Mucilaginous Acaciæ | ... | ʒij. |
| Aquam | ad | ℥iv. |

Put the mucilage into a mortar, stir it round a little so as to get some on the sides and on the pestle, then add the oil in a slow stream, stirring lightly all the time. Triturate for two minutes or so, until the mixture appears quite uniform, then add about a drachm of water, and stir until uniform and creamy; then add further small quantities of water, mixing each addition until the product is quite uniform before the next is poured in; as the process proceeds larger quantities of water can be added at once until the emulsion is quite thin and milky, when it may be transferred to the bottle, and the rest of the water added there. In this example the amount of mucilage is larger than the minimum necessary, the aim of most dispensers being to add as little as possible of gum or other similar agent.

Generally speaking, for the ordinary fixed oils, 1 part of gum is necessary for 4 parts of oil; for practical purposes 1 part of gum may be taken as equivalent to 2 parts of mucilage, so that 1 part of mucilage is sufficient for 2 parts of oil. The above prescription will therefore become either

| | | |
|---|---|---|
| ℞ Olei Amygdalæ | ... | ʒiij. |
| Pulveris Acaciæ | ... | gr. xlv. |
| Aquam | ad | ℥iv. |

or

| | | |
|---|---|---|
| ℞ Olei Amygdalæ | ... | ʒiij. |
| Mucilaginis Acaciæ | ... | ʒiss. |
| Aquam | ad | ℥iv. |

If the powdered gum be used this may be rubbed up in a mortar with about 45 minims of water, allowing sufficient time for it to become dissolved, and then proceeding as before. This is the safest plan. Another method, preferred by some, is to rub the dry powdered gum with the oil until thoroughly mixed, then to add $1\frac{1}{2}$ drachms of water in one quantity, stirring well till quite creamy; the mucilage and the emulsion are thus made simultaneously, but it takes practice to do this well. It must be remembered that gum acacia dissolves somewhat slowly, and that in the second method it is delayed by the oil from coming into intimate contact with the water; this stage of the process must not be hurried therefore. When the product is quite creamy and homogeneous in appearance, more water is added in small quantities, care being taken that each addition is perfectly mixed in before the next is made.

It is well for those who are not already adept at making emulsions to try the first of the above prescriptions as written, and then to try the effects of using only 2 drachms and $1\frac{1}{2}$ drachms of mucilage respectively, repeating the attempt if necessary until a good result is obtained with the $1\frac{1}{2}$ drachms. The quality of the gum is important, as well as the freshness of the mucilage; an inferior sample of gum will make the emulsion very much more difficult of preparation. The second prescription should also be tried by each of the methods described, and all the emulsions so made should be put aside and compared after several days' standing.

The examples so far considered contain nothing but the constituents of the emulsion—that is to say, the two immiscible liquids, in this case oil and water, and the emulsifying agent gum. As a rule, however, some other substance or substances are included in the same prescription with these, and such additional ingredients may have a considerable influence on the emulsion. This influence is usually in the direction of making the emulsion more troublesome to prepare, or more liable to separate on keeping. The following are common examples of such mixtures:—

| | | |
|---|---|---|
| ℞ Olei Ricini | ... | ʒiij. |
| Mucilaginis Acaciæ | ... | ʒij. |
| Syrupi | ... | ʒjss. |
| Tincturæ Zingiberis | ... | ʒss. |
| Aquam | ad | ℥iij. |

| | | |
|---|---|---|
| ℞ Copaibæ | ... | ℥ss. |
| Pulveris Acaciæ | ... | ʒij. |
| Spiritus Etheris nitrosi | ... | ʒiij. |
| Potassii Chloratis | ... | gr. xxxvi. |
| Aquam Chloroformi | ad | ℥vj. |

Generally speaking, either alcoholic preparations, such as tinctures and spirits, or solutions of salts, are hostile to the emulsion; we must here follow the rule already given of keeping such ingredients as far apart as possible, and diluting before mixing. The emulsion should be made with the essential ingredients first, most of the vehicle being added, and this part of the mixture transferred to the bottle; the salt should then be dissolved in, or the tincture diluted with, the remainder of the vehicle, keeping from one-sixth to one-quarter for this purpose, additions being then made to the emulsion with gentle shaking after each addition.

Salts which are alkaline in reaction are usually rather favourable than otherwise to an emulsion; the alkali saponifies a small portion of the oil, or combines with a small portion of the resin, in either case making a soap which assists emulsification. Some emulsions are prepared with alkali only; we shall deal with these later.

In the first stage of making an oil emulsion the consistence of the mixture is of considerable importance. If oil and mucilage of gum acacia are triturated together in a mortar a stiff, sticky mixture results; in such a medium each globule of oil that comes under the pestle is broken or drawn out into smaller globules; but if the mucilage had been previously diluted the medium would not offer enough resistance to the globules, and they would slip away from the pestle unbroken, so that a poor emulsion or a complete failure would result. On the other hand, the medium must not be so stiff as to resist proper mixing; on gradually adding oil to mucilage the mixture in many cases becomes too stiff, and it must then be thinned by adding a little water. For this reason it is often necessary to add alternately small quantities of oil and of water, mixing well after each.

